# A89 PREVALENCE AND OUTCOMES OF SERRATED POLYPOSIS SYNDROME IN TERTIARY GASTROENTEROLOGY PRACTICES: A RETROSPECTIVE ANALYSIS

**DOI:** 10.1093/jcag/gwae059.089

**Published:** 2025-02-10

**Authors:** S X Jiang, A Zarrin, E Taylor, P Tavakoli, S Bell, A Walia, S Pang, M Rui Xuan Yu, D Motomura, E Lam, R Enns, J Telford, W Xiong, N Shahidi

**Affiliations:** The University of British Columbia Faculty of Medicine, Vancouver, BC, Canada; The University of British Columbia Faculty of Medicine, Vancouver, BC, Canada; StPaul’s Hospital, Division of Gastroenterology, Vancouver, BC, Canada; StPaul’s Hospital, Division of Gastroenterology, Vancouver, BC, Canada; The University of British Columbia Faculty of Medicine, Vancouver, BC, Canada; StPaul’s Hospital, Division of Gastroenterology, Vancouver, BC, Canada; StPaul’s Hospital, Division of Gastroenterology, Vancouver, BC, Canada; The University of British Columbia Faculty of Medicine, Vancouver, BC, Canada; StPaul’s Hospital, Division of Gastroenterology, Vancouver, BC, Canada; StPaul’s Hospital, Division of Gastroenterology, Vancouver, BC, Canada; StPaul’s Hospital, Division of Gastroenterology, Vancouver, BC, Canada; StPaul’s Hospital, Division of Gastroenterology, Vancouver, BC, Canada; StPaul’s Hospital, Department of Pathology and Laboratory Medicine, University of British Columbia, Vancouver, BC, Canada; StPaul’s Hospital, Division of Gastroenterology, Vancouver, BC, Canada

## Abstract

**Background:**

While serrated polyposis syndrome (SPS) is associated with an increased risk of colorectal cancer (CRC), incident CRC can be mitigated through programmatic endoscopic surveillance. Diagnosing SPS remains challenging due to physician awareness and the logistics of cumulative polyp tracking. There is a lack of data on SPS prevalence and outcomes outside of established cohorts.

**Aims:**

To evaluate the prevalence and outcomes of SPS within tertiary gastroenterology practices.

**Methods:**

Using a validated histopathology database, all patients with at least 1 sessile serrated lesion (SSL) removed by 1 of 11 gastroenterologists in a tertiary centre between 2013-2023 were considered for evaluation. All patients were subsequently screened for SPS based on the 2019 World Health Organization (WHO) criteria. Patients meeting SPS criteria underwent full chart review for demographic, procedural, and clinical outcomes. Analysis was based on serrated class lesions (SCLs), which included sessile serrated lesions, hyperplastic polyps, and traditional serrated adenomas.

**Results:**

A total of 6117 patients had at least 1 SSL of which 178 (3%) met SPS criteria (110 WHO I, 10 WHO II, 58 WHO I/II). Median age at first colonoscopy was 62 years (IQR 54-71 years) and 115 (65%) were female. Diagnostic criteria were achieved over a median of 3 years (IQR 1-9 years) and a median of 3 colonoscopies (IQR 2-4 colonoscopies). On index colonoscopy, patients eventually meeting SPS criteria had a median of 3 SCLs (IQR 2-6 SCLs) with median largest lesion size of 11mm (IQR 5-18mm) whereas patients without SPS had a median of 1 SCL (IQR 1-2 SCLs) with median largest lesion size of 5mm (IQR 4-8mm) (p<0.001). SPS was diagnosed by the gastroenterologist in only 132 (74%) patients. SPS was managed by endoscopy and surgery in 157 (88%) and 21 (12%) respectively. Reasons for surgery included polyp burden (9, 5%), endoscopic adverse event (1, 0.5%) and CRC (11, 6%). CRC was diagnosed either before or concurrent to SPS diagnosis in 7 (63.6%).

**Conclusions:**

In patients with at least 1 SSL, the prevalence of SPS was 3% and the diagnosis was recognized in 74% by the primary gastroenterologist. Increased awareness of SPS criteria alongside consideration for modified surveillance intervals based on index SCL size and number may improve patient outcomes.

**Funding Agencies:**

**None**

